# Development of a sticker sealed microfluidic device for *in situ* analytical measurements using synchrotron radiation

**DOI:** 10.1038/s41598-021-02928-2

**Published:** 2021-12-08

**Authors:** Itamar T. Neckel, Lucas F. de Castro, Flavia Callefo, Verônica C. Teixeira, Angelo L. Gobbi, Maria H. Piazzetta, Ricardo A. G. de Oliveira, Renato S. Lima, Rafael A. Vicente, Douglas Galante, Helio C. N. Tolentino

**Affiliations:** 1 Brazilian Synchrotron Light Laboratory (LNLS), Brazilian Center for Research in Energy and Materials (CNPEM), Campinas, 13083-970 Brazil; 2 Institute of Chemistry, Federal University of Goiás, Campus Samambaia, Goiânia, 74690-900 Brazil; 3 Brazilian Nanotechnology National Laboratory (LNNano), Brazilian Center for Research in Energy and Materials (CNPEM), Campinas, 13083-970 Brazil; 4 Institute of Chemistry, University of Campinas, Campinas, SãoPaulo 13083-970 Brazil

**Keywords:** Electrochemistry, Analytical chemistry, Microfluidics, Characterization and analytical techniques, Imaging techniques, Design, synthesis and processing

## Abstract

Shedding synchrotron light on microfluidic systems, exploring several contrasts *in situ*/*operando* at the nanoscale, like X-ray fluorescence, diffraction, luminescence, and absorption, has the potential to reveal new properties and functionalities of materials across diverse areas, such as green energy, photonics, and nanomedicine. In this work, we present the micro-fabrication and characterization of a multifunctional polyester/glass sealed microfluidic device well-suited to combine with analytical X-ray techniques. The device consists of smooth microchannels patterned on glass, where three gold electrodes are deposited into the channels to serve *in situ* electrochemistry analysis or standard electrical measurements. It has been efficiently sealed through an ultraviolet-sensitive sticker-like layer based on a polyester film, and The burst pressure determined by pumping water through the microchannel(up to 0.22 MPa). Overall, the device has demonstrated exquisite chemical resistance to organic solvents, and its efficiency in the presence of biological samples (proteins) is remarkable. The device potentialities, and its high transparency to X-rays, have been demonstrated by taking advantage of the X-ray nanoprobe Carnaúba/Sirius/LNLS, by obtaining 2D X-ray nanofluorescence maps on the microchannel filled with water and after an electrochemical nucleation reaction. To wrap up, the microfluidic device characterized here has the potential to be employed in standard laboratory experiments as well as in *in situ* and *in vivo* analytical experiments using a wide electromagnetic window, from infrared to X-rays, which could serve experiments in many branches of science.

## Introduction

Microfluidic devices operate with small amounts of samples through micrometer and/or sub-micrometer channels^1^. As the main characteristics, these devices show reduced size, low weight, and high throughput. These characteristics have attracted tremendous and widespread attention by the synchrotron community^2,3^ interested in compatible instrumentation for *in situ* and *in vivo* experiments. To date, reported applications of these devices on synchrotrons, involving studies on protein crystallography^4,5^, advanced materials^6^, electrochemistry^7,8^, catalysis^9–11^, *in vivo* experiments (eukaryotic and bacteria cells)^6,12,13^, established a paramount experimental contribution for drawing a picture of these complexes systems, providing precious information about electronic, structural, and chemical properties in real-time, *in situ* and/or operando. However, developing a small, versatile, and compact microfluidic device able to operate in synchrotron beamlines, especially at nanoprobes where the X-ray beam size is only hundreds of nanometers, has remained a challenge due to sample environment requirements. Arguably, limits should be established on such devices. It is crucial to define what is the good compromise between the selected material and the reliability of the microfabrication process when the device is to be applied in experiments that cover a wide window of the electromagnetic spectrum (X-rays, Infrared or visible light)^14^.

In the last two decades, poly(dimethylsiloxane), commonly called PDMS (an elastomer), has been the most applied material as substrate and sealing layers in microfluidic devices^15–17^ due to its remarkable physical and chemical properties such as biocompatibility, flexibility, transparency, and low toxicity^1,18,19^. Nevertheless, PDMS presents some disadvantages that should be considered: it is permeable to small molecules due to its porosity, which can affect the devices throughput^20^, and it shows low chemical resistance to many organic solvents^21^. Hence, this elastomer can lead to non-accurate results in biological assays and it is restricted to analyses in aqueous media. Similar limitations are also found in poly(methyl methacrylate) (PMMA), another popular polymer substrate for the production of microfluidic chips. In addition, PDMS devices might reach millimeters of thickness, which is non-compatible with X-rays measurements due to the lower transmission coefficient for X-rays when compared to commonly applied polymers in microfluidic. For example, a millimeter thick cyclic olefin copolymer (COC) based device attenuates, at 12.4 keV, seven-fold less than PDMS^22^, being more discrepant at lower energies. A similar proportion is observed by comparing polyester to PDMS.

An insightful approach to minimize the drawbacks mentioned before, consists in drastically changing the sealing process by selecting a sealing layer transparent to X-rays and chemically resistant, which leads to well known polymer films such as polyester, polypropylene, polyamide, and polycarbonate (Supporting Information). In addition, few works have proposed using ultraviolet (UV) sensitive adhesive, like the Norland Optics Adhesive (NOA), as substrate or adhesion layer^23–26^ in microfluidic devices. NOA is a thiolene based polymer that attracted attention due to exhibiting interesting properties like low cost, optical transparency in the visible range, high chemical resistance to strong solvents (acetone, hydrochloric acid, toluene)^24,27^, and hydrophobicity^26^ becoming a great candidate for applications in synchrotron beamlines^28,29^.

Motivated by the relevance and impact of synchrotron studies for diverse areas as well as by the challenges in manufacturing microfluidic devices for such applications, we have developed the microfabrication of a three-electrode microfluidic device based on a new sealing method, which is compatible with X-rays (in reflection mode), infrared, and visible light. The device has been designed with only two main parts; a glass substrate, and a thin polymer employed as a sealing layer. We have used a UV-sensitive adhesive to promote adherence between polyester and glass. It is worth noting that the microfabrication involves well-stablished and scalable techniques and the resulting device presents satisfactory bonding strength and high chemical stability in both organic and biological media. The approach adopted in this work converged to a multifunctional microfluidic device.

## Results and discussions

### Microfabrication procedure

Initially, 3 cm $\times$ 3 cm glass squares were cut, cleaned up using piranha solution at a temperature of 60 $^{\circ }$ C, and then used as a substrate. The microchannels were prepared in a clean room using a geometry defined by the photolithography mask. In total, four lithography masks or channel geometry, were prepared: 100 $\mu$m (C1) and 100 $\mu$m plus a reservoir (res) with 300 $\mu$m (C2), 200 $\mu$m (C3), and 200 $\mu$m plus a reservoir(res) with 600 $\mu$m (C4). Prior to the microchannel patterning, a layer of 60 nm of chromium was evaporated on the clean glass, working as a protective layer that improves the isotropic corrosion of the glass. To prepare the channels, the trenches were exposed to a wet chemical etching^30^ using a solution at HF 49%, NH_4_F 1.38 M, and HCl 38%, and hydrofluoric acid (HF) 20%, resulting in smooth channels. After the glass corrosion, using a second photolithography mask, three electrodes were patterned, and then gold was deposited on the channels by magnetron sputtering. After the deposition of the three gold electrodes, the device was drilled to enable connecting the inlet and outlet tubes. The sequence of the channel and electrode preparation is shown in Fig. S1 (Supporting Information).

**Figure 1:**
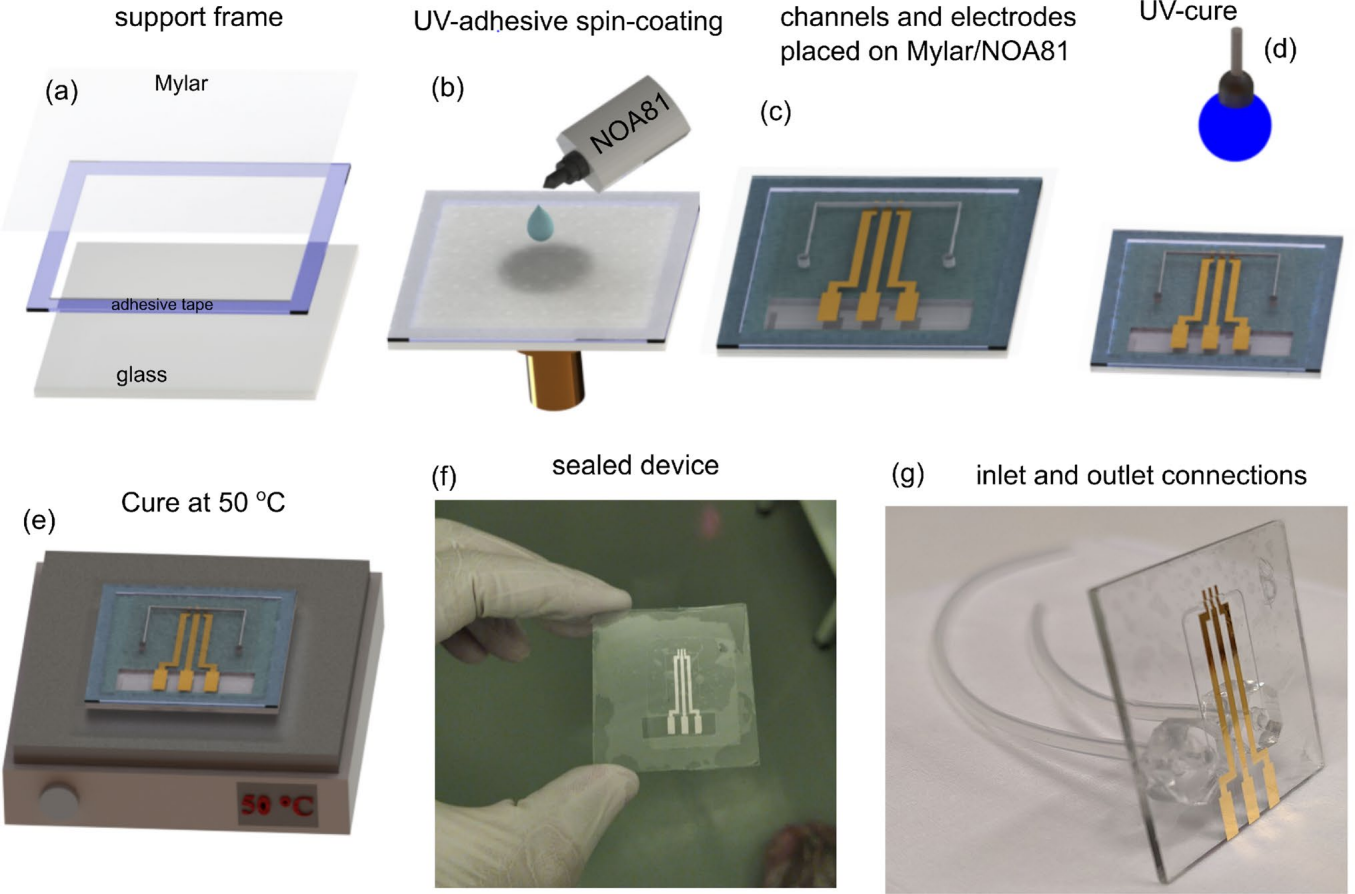
Sequence of the sealing process (**a**) Glass frame used as a support for the polyester film (**b**–**c**) Spin-coating process of the NOA on the polyester film, and the procedure to attach the device to the “stikcker” (**d**–**e**) UV-cure (2 min) and the thermal annealing at 50 $^{\circ }$ C (12 h) employed to improve the adhesion between the device and sealing layer (**f**–**g**) Shows the device completely sealed, and after bound the inlet and outlet connections, respectively.

The most important and delicate step of this work is the sealing process, once the sealing layer is a thin film. To succeed, an adhesive composed of NOA and a polyester film was prepared, herein this stack is also called *sticker*, which simplifies the sealing process. The sealing steps are summarized in Fig. 1. Firstly, a polyester film with 12 $\mu$m of thickness was fixed with double-face adhesive tape on a glass frame to ensure mechanical stability (Fig. 1a). Thus, the stack glass-frame/polyester was transferred to the spin-coater and a thin film (up to 1 $\upmu$m) of NOA was prepared on the polyester film at 5000 rpm for 35 s. Then, the device was attached to the NOA side as shown in Fig. 1b–c. Finally, a cure was done exposing the device to ultraviolet light ($\lambda$=365 nm) for 2 min at room temperature. After the curing step, the device was kept at 50 $^{\circ }$ C for 12 h to improve the adhesion (Fig. 1d,e). In order to access the three gold electrodes, the spare piece of the polyester film was cut-off from the sealed device (Fig. 1f). In the last step, the glass was drilled in the backside, and the inlet and outlet connections (silicone tubes) were welded using PDMS support, which gives high mechanical stability to the connections. All the sealing steps were carried out into a cleanroom.

### Inspection of the channels and electrodes

**Figure 2:**
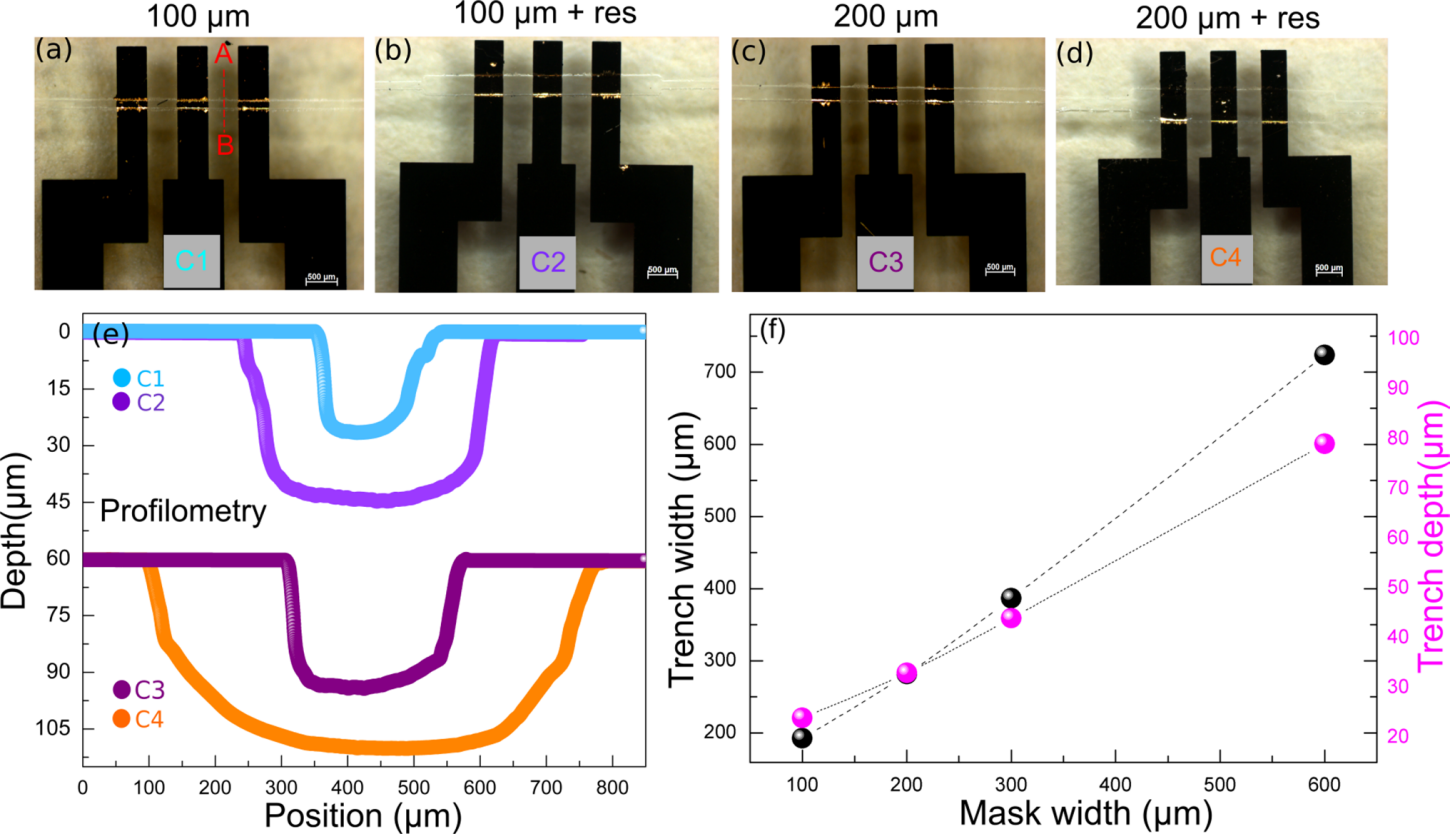
Characterization of the channel width and gold electrodes. (**a**–**d**) Optical microscopy of all the devices designed (C1-100 $\mu$m, C2 $\mu$m+res, C3 200 $\mu$m, and C4) 200 $\mu$m+ res. (**e**) Profilometry measurements showing the channel depth and width. The red line A–B corresponds to the line where the measurements were taken (**f**) Dependence of the trench width and trench depth with the lithography mask width.

Using glass as a substrate for microfluidic devices has some advantages: glass is inert to organic solvents, supports high temperatures, and might exhibit biocompatibility. However, the corrosion process could lead to microcracks, microtextures, and an enlargement of the channel due to its isotropic corrosion mechanism. Thereby, prior to the sealing process, optical microscopy and profilometry were employed to investigate how precise the glass corrosion was. As it can be seen in Fig. 2, the micrographs, from (a) to (d), show small defects on the channel border, which had no impact on the sealing process. In addition, concerning the channel geometry, it can be observed a channel patterning with high resemblance to the lithography mask, pointing out the good precision of such process. It is also possible to observe that the gold electrodes do not exhibit any large discontinuity or faults. In order to confirm the uniformity of the electrodes, the electrical resistance was measured for all devices, which revealed an average of 28 ± 1 $\Omega$, a value acceptable for gold thin films with about 100 nm of thickness^31^. Nevertheless, profilometry along the A-B line profile for all cells revealed channels with a slight deviation in width from the original lithography mask, as shown in Fig. 2e–f. Usually, such deviation is due to the high intrinsic isotropicity of the glass liquid-phase etching. In addition, the channel depth exhibited a linear behavior following the mask width, as shown in Fig. 2f, hence the etching rate is significantly different in both dimensions.

### Sealing strength by bursting pressure and electrochemistry tests

For the new sealing process proposed in this work, which combines a sub-micron layer of UV-adhesive and a ten of micron of the sealing layer, it is expected an inherent limit pressure (flow rate threshold) denominated bursting pressure. Hence, the bond strength is a fundamental and critical parameter in microfluidic devices where the burst pressure strongly depends on the surface energy of the liquid-interface, wettability, and channel geometry. Initially, to check the efficiency of the sealing process, a syringe pump was used to inject water at 50 $\mu$L/min, and the flow through the channel was observed placing the device on an optical microscope (see video Supporting Information). Yet, no leak was observed, which revealed devices completely sealed (See the video in the supplementary material) and arguably ready to operate.

**Figure 3:**
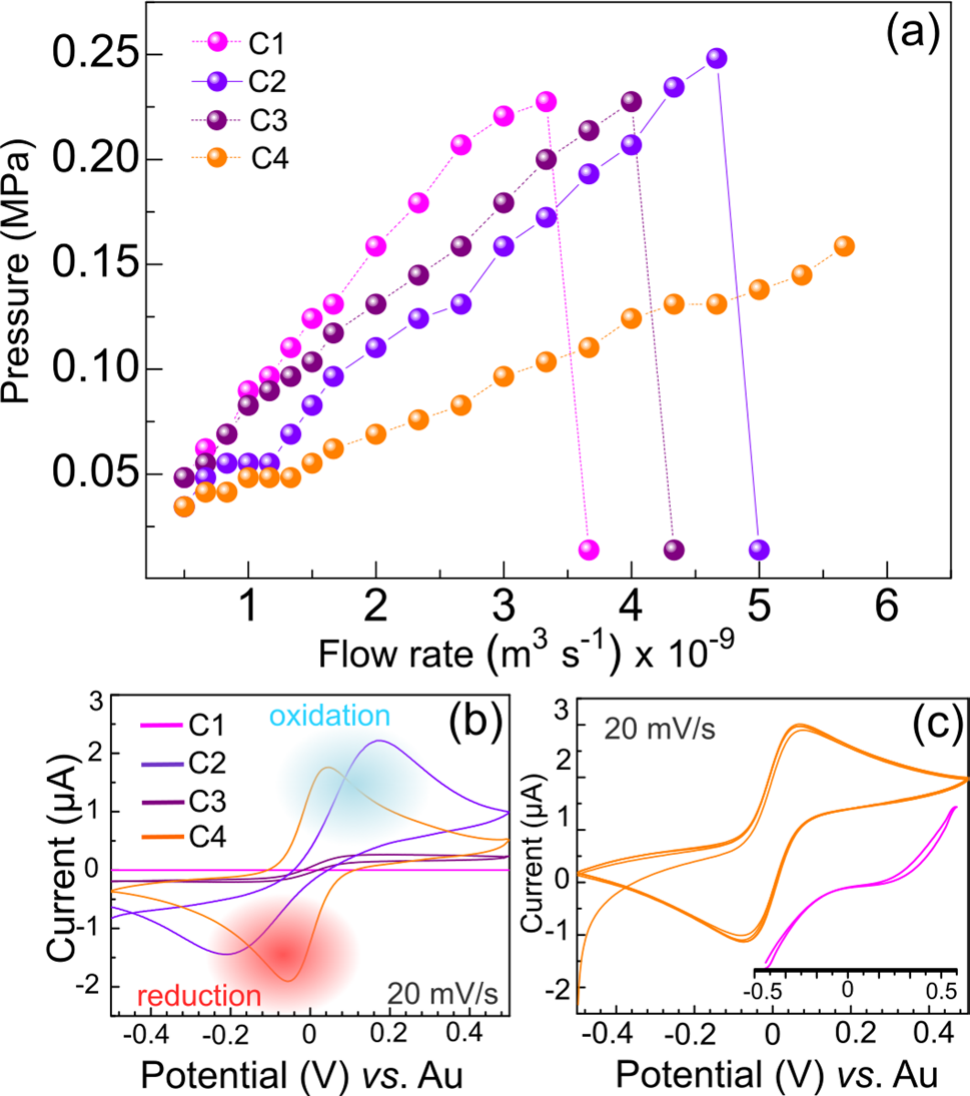
Pressure tolerance and electrochemical tests. (**a**) Determination of the burst pressure for the devices. At each flow rate, the pressure required is obtained using an HPLC pump. The pressure limit (bursting) is clearly observed for channels with 100 $\mu$m (C1), 100 $\mu$m+reservoir (C2), 200 $\mu$m (C3). Intentionally C4 (200 $\mu$m+reservoir) was kept sealed. (**b**) Cyclic voltammetry obtained at 20 mV/s (**c**) electrochemical stability of the device accessed through cyclic voltammetry by varying the potential from −0.5 to 0.5 V (three cycles). For clarity the voltammetry for C1 is zoomed and shown as an inset in (**c**).

In order to determine the burst pressure, deionized water was pumped into the microchannels at about 25 $^{\circ }$ C. The water was driven through the channel using high-performance liquid chromatography (HPLC) pump whilst the same pump gave the pressure for each inlet flow rate, while the outlet was kept open to the atmosphere. In Fig. 3a it is shown the results of pressure measurements for the four devices geometry (C1, C2, C3, C4). The volumetric flow rate was varied from 30 to 340 $\mu$l/min. As it can be seen in Fig. 3, the measurements reveal a burst pressure at about 0.22 MPa (or 33 psi), clearly identified due to drastic pressure drops for C1, C2, and C3. The C4 device has the larger reservoir (600 $\mu$m) and was intentionally preserved because the flow rate achieved exceeded our requirements.

In microfluidic devices the pressure versus flow rate is governed by the Hagen–Poiseuille’s law^32^ Q=${\hbox {R}_{\mathrm{H}}}$.$\delta$P, where Q is the volumetric flow rate, $\delta$P is the pressure difference, and ${{\hbox {R}}_{\mathrm{H}}}$ is the hydrostatic resistance of the device, which is inversely proportional to the channel cross-section area as described elsewhere^32^. Therefore, it is expected linear dependence of the pressure with the volumetric flow rate in sealed devices, where the slope gives the hydrostatic resistance of the channel geometry. Hence, narrower channels lead to higher ${{\hbox {R}}_{\mathrm{H}}}$ values, as shown in Fig. 3.

The nature of the interface is an important characteristic interfering on the sealing process, limiting the maximum flow rate (or pressure) through the device. For example, it is found bursting pressure varrying from 0.096 to 0.94 MPa for PDMS/glass device, reaching to 4.5 MPa in PDMS/PDMS devices^18,33–35^. On the other hand, mediated by NOA, a high pressure tolerant (12 MPa) device was obtained in glass-polymer-glass^36^. For comparison, in this work the polyester/glass device tolerated a pressure of 0.22 MPa, unfortunately, no data about the sealing interface described here was found for a comparison.

To investigate the performance of the microfluidic device in electrochemistry experiments and accessing its electrical measurement functionality, as designed, the reversible redox reaction ${\hbox {Fe}^{\mathrm{3+}}}/{\hbox {Fe}^{\mathrm{2+}}}$ was characterized by cyclic voltammetry(CV) at scan rate of 20 mV/s from 0.5 V to -0.5 V. Prior to the CV experiments, the channel was filled with an electrolyte consisting of 10 mmol ${{\hbox {L}}^{\mathrm{-1}}}$ of potassium ferricyanide ($\hbox {K}_{3}[\hbox {Fe}(\hbox {CN})_{\mathrm{6}}]$) and potassium ferrocyanide (${\hbox {K}_{\mathrm{4}}[\hbox {Fe(CN)}_{\mathrm{6}}]}$) supported in 0.1 mol ${{\hbox {L}}^{\mathrm{-1}}}$ of potassium chloride (KCl). The potential was swept using the central electrode as a working electrode, the right electrode as a reference electrode, and the left electrode as a counter electrode. Figure 3b shows the electrochemistry response for the devices. The CV shape changes from C1 to C4, exhibiting prominent oxidation (positive) and reduction (negative) current peaks for C2 and C4; the two cells with a reservoir. The transient voltammograms typical of macroelectrodes were reached by both the electrodes, reflecting a semi-infinite linear diffusion profile^37^. The average peak-to-peak separations were found to be lower than 90.0 63 mV^38^, implying the occurrence of reversible systems that are likely because of the high conductivity and active area of electrodes, which favor the charge-transfer kinetics. In addition, for C1 (see inset in Fig. 3c) and C3 the sigmoidal-shaped voltammetric responses are characteristics of ultramicroelectrodes by exhibiting steady-state currents driven by radial diffusion^37^. Theses systems are preferred for the ultrasensitive affinity-based and enzymatic detection of biomolecules due to their higher signal-to-noise ratio when compared with electrodes bearing linear diffusion dominant behavior^39^. In complement, Fig. 3c shows a continuously cycling using the cell C4, where it is observed a stable response over the three cycles measured.

In this sense, the approach employed in this work imposes to the electrodes a curved geometry on the channel, as a consequence, for each cell the volume and the electrolyte-electrode contact area are different. Summarizing, small channels, even on the reservoir, have a smaller volume and consequently small exposed areas. Therefore, the results obtained from the electrochemistry measurements are in agreement with studies related to electrochemistry using microelectrodes, where it is described the dependence of the CV hysteresis (or shape) with the diffusion zones geometry close to the electrode surface^40^. Our findings are also important to evaluate the microfabrication procedure in terms of contamination due to the microchannels to be attached to the NOA/polyester sticker during the sealing process. Thus, the presence of the redox peaks in the CV indicates that the NOA does not cover the electrodes.

### Chemical resistance in organic solvents and biological fluid

**Figure 4:**
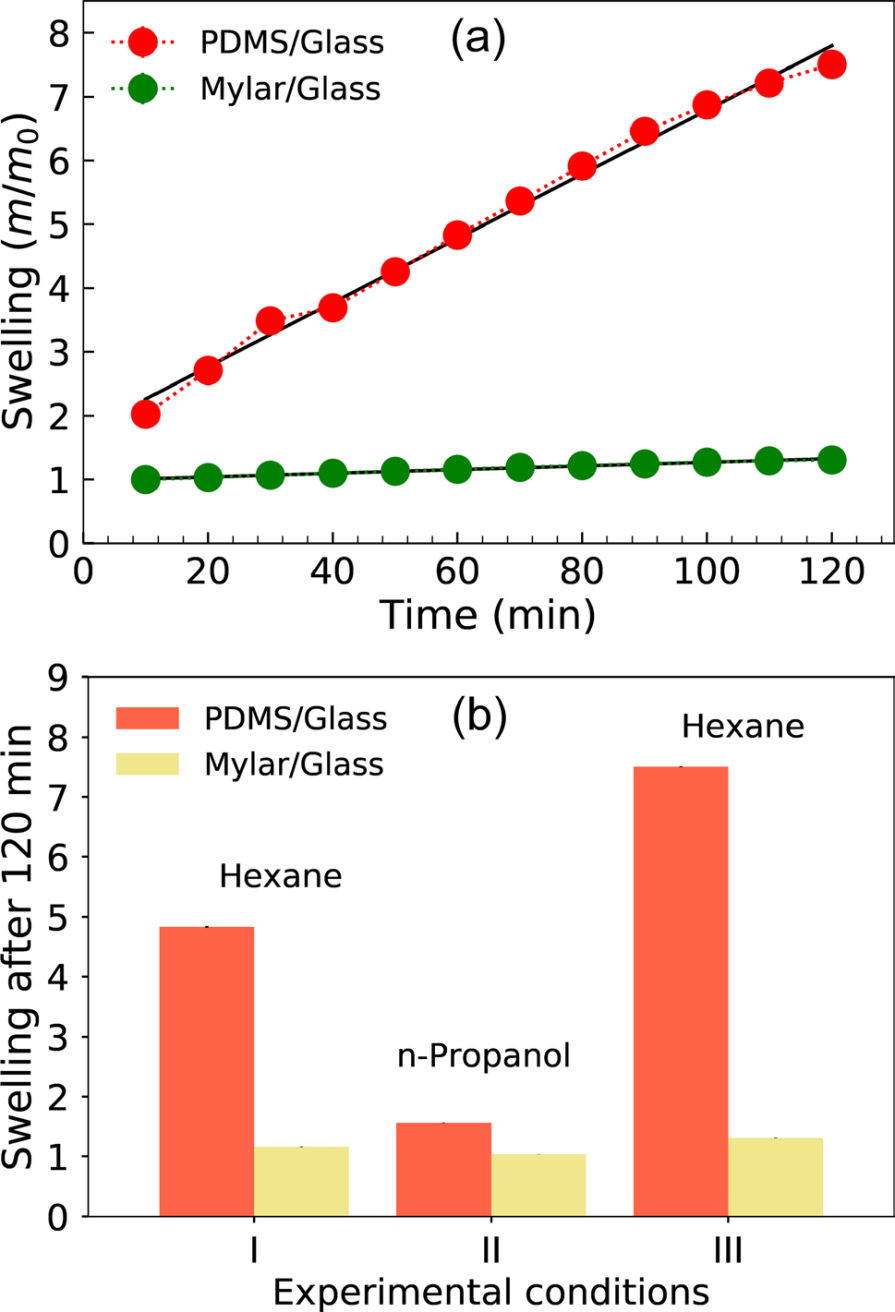
Values of S for the PDMS/glass and polyester/glass devices. (**a**) Data obtained after exposition to hexane for 120 min and (**b**) specific results associated with expositions for 60 (I,II) and 120 min (III) to hexane and n-propanol as highlighted, the error bars mean confidence intervals for $\alpha$ = 0.05 (*n* = 10).

To investigate the chemical stability of the polyester-glass devices, those chips were compared to the PDMS-glass chip by measuring the swelling ratio in the presence of organic solvents (hexane and n-propanol). For these tests, the PDMS based devices were fabricated as described in the literature^41–43^. Briefly, channels (500 $\upmu$m-width and 30 $\upmu$m-depth) were engraved on PDMS by soft lithography, whereas the bonding step with a planar slide of glass was based on oxidation by an oxygen plasma. In practice, each solvent was pumped into the devices at a flow rate of 50.0 $\mu$L ${\hbox {min}^{\mathrm{-1}}}$. The mass of the filled devices (C4) was continuously monitored. The values of the swelling parameter (S=$\frac{m}{m_0}$) were calculated by dividing the gradually measured masses (m) by the reference or initial data, i.e., the mass recorded right after filling the device with the solvent (${{\hbox {m}}_{\mathrm{0}}}$)^44^. Assuming a linear fit, the PDMS-glass device shows a swelling rate of 5.03 $\times$ ${\hbox {10}^{\mathrm{-2}}}$ ${{\hbox {min}}^{\mathrm{-1}}}$ (${{\hbox {R}}^{\mathrm{2}}}$ = 0.98) in the presence of hexane as depicted in Fig. 4a. Conversely, this rate was only 2.88 $\times {\hbox {10}^{\mathrm{-3}}} {{\hbox {min}}^{\mathrm{-1}}} ({{\hbox {R}}^{\mathrm{2}}} = 0.99)$ for the polyester-glass chip. According to Fig. 4b, S was measured as 7.51 ± 0.01 (PDMS-glass) and 1.31 ± 0.01 (polyester-glass) after an exposition to hexane for 120 min. In addition, the swelling ratios in the presence of n-propanol for 60 min were 1.56 ± 0.01 (PDMS-glass) and 1.03 ± 0.01 (polyester-glass). These results suggest our device provides high chemical resistance to organic media. It is noteworthy that the polyester-glass chips described herein have met important requirements aforementioned, demonstrating a swelling of 17-fold lower than similar devices based on PDMS/glass.

To explore the multifunctional characteristic of the polyester-glass device, its chemical inertia was probed in the presence of biological samples (under flow) as well. Conversely to the observed for PDMS-glass devices, our mylar-glass chip further exhibited low adsorption of proteins. In practice, protein-enriched solution of bovine serum albumin (BSA) 10.0 mg ${{\hbox {mL}}^{\mathrm{-1}}}$ in potassium chloride (KCl) 1.0 ${{\hbox {mol L}}^{\mathrm{--1}}}$ was pumped into the devices for 2 h at 10.0 $\mu$L ${{\hbox {min}}^{\mathrm{-1}}}$. Such protein was chosen as biomolecule model by accounting for  60% of the total proteins in blood plasma^37^. Afterwards, the protein concentrations were quantified through a conventional method, i.e., Bradford assays, which were performed according to the official protocol using spectrophotometer and 96-well polystyrene plates (Kasvi, model K30-5096P)^45^. The protein concentrations were determined as 8.1 ± 0.8 mg ${\hbox {ml}^{\mathrm{-1}}}$ (*n* = 3) and 9.8 ± 0.9 ${{\hbox {ml}}^{\mathrm{-1}}}$ (*n* = 3) for the samples exposed to the PDMS-glass and mylar-glass devices, respectively. While the original protein concentration was decreased by 19% in the first case, the latter chip resulted in a percentage drop of only 2%. These tests were once again performed on the polyester/glass device type C4.

### Nanofluorescence imaging on the microfluidic device

Carnaúba is a state-of-the-art X-ray nanoprobe beamline in commissioning at the SIRIUS/LNLS^46^. Its main characteristic is to provide a fully coherent X-rays beam (2.05 to 15 keV) with spot on the sample varying from 30 to 600 nm, depending on the experimental station, allowing studies using cutting-edge X-rays-based techniques at nanoscale. One of the prerequisites for *in situ* experiments at Carnaúba is to work with a small amount of samples (solid or liquid) and optimized sample holders, and as previously mentioned, microfluidic devices have risen as a great solution for complexes sample environments.

**Figure 5:**
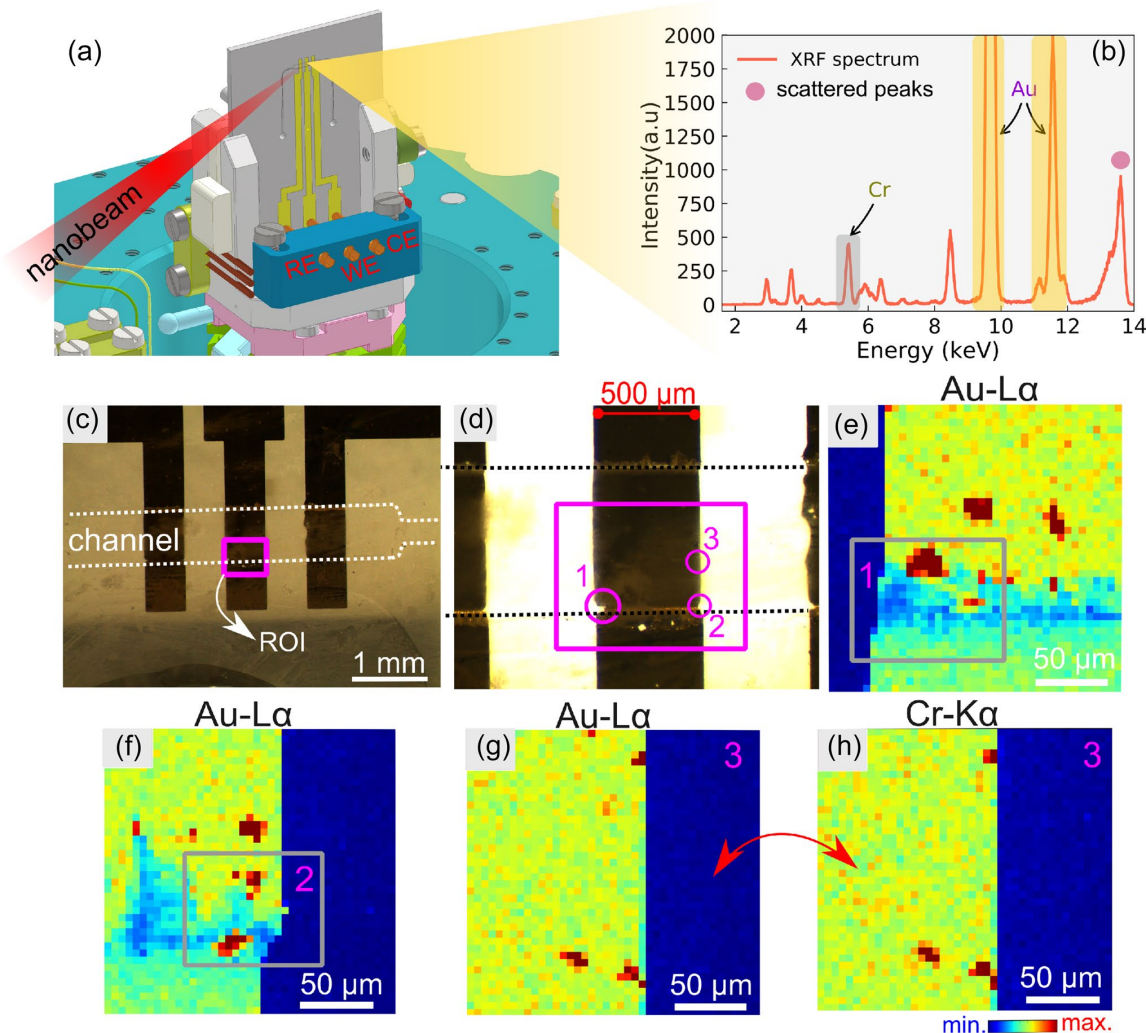
Nanofluorescence experiments on the microfluidic device. (**a**) Represents a 3D scheme of the microfluidic sample holder. (**b**) Fluorescence spectrum collected at the central electrode. (**c**–**d**) Optical micrography of the channel and electrodes highlighting the ROI (magenta rectangle) scanned by a nanobeam of about 600 nm $\times$ 600 nm. (**e**–**f**) Show the Au-$L_{\alpha }$ (9713 eV) fluorescence maps about the circle 1 and 2 on the electrode border. (**g**–**h**) Au ($L_{\alpha }$), and Cr-$K_{\alpha }$ (5.4 keV) maps obtained at the circle 3. The experiments were performed on the channels filled with water. The nano-XRF maps have a pixel size of 5 $\mu$m and an entire acquisition time of 65 s (22.5 ms per pixel or step).

The transparency of the device to synchrotron X-rays, essential to advance in experiments involving the techniques mentioned above, was tested acquiring 2D nano-XRF images on the central electrode (WE) by scanning the microfluidic device (Fig. 5a) about the nanobeam. The experiments were carried out at the end-station Tarumã at the Carnaúba beamline. A Kirkpatrick-Baez (KB) mirror setup focused the X-ray beam at 600 nm $\times$ 600 nm with a primary beam intensity of ${{\hbox {10}}^\mathrm{9}}$ photons/second. Normal focusing conditions are about 200 nm $\times$ 500 nm, but the device was on purpose out of focus for this experiment. The incidence angle was set to 80$^{\circ }$, almost frontal, to maximize the collection of XRF photons (Fig. 5b) for all nano-XRF experiments. The X-ray energy was selected by a four-bounces Si(111) crystal monochromator providing a resolution of ${\hbox {10}^{\mathrm{-4}}}$. An optical micrography of the device, as mounted in the experimental station, is shown in Fig. 5c. The magenta rectangle indicates the region of interest (ROI) from where the nanofluorescence maps were acquired. Zooming over the ROI reveals micro faults in the right and left sides of the electrode (Fig. 5d).

The excitation energy was set to 13.657 keV (9.748 keV) to optimize the fluorescence counts for Au (Cr) (Fig. 5b). Chromium is the gold’s adhesion buried layer with 30 nm of thickness. Prior to the experiment, the channel was filled with water. The nano-XRF maps for Au-$L_{\alpha }$ displayed in Fig. 5e, f were acquired in continuous mode (flyscan) scanning an area of 200 $\times$ 200 $\mu$m with 5 $\mu$m step and 22.5 ms counting time per step over circles 1 and 2. Full maps last 65 s and elemental contrast is obtained by selecting the corresponding energy range on the integrated spectrum over the scanned area (highlighted in Fig. 5b). The maps reveal small cracks or micro faults as observed by optical microscopy. Additionally, some very intense spot-like features come from the lift-off process due to a few flakes of gold/chromium on the electrode surface. Figure 5g–h shows fluorescence maps obtained into the channels at the circle 3 as indicated. As the main conclusion, the maps clearly show a high contrast on the gold Au-$L_{\alpha }$ and on the Cr-$K_{\alpha }$, despite the lower energy of the latter.

**Figure 6:**
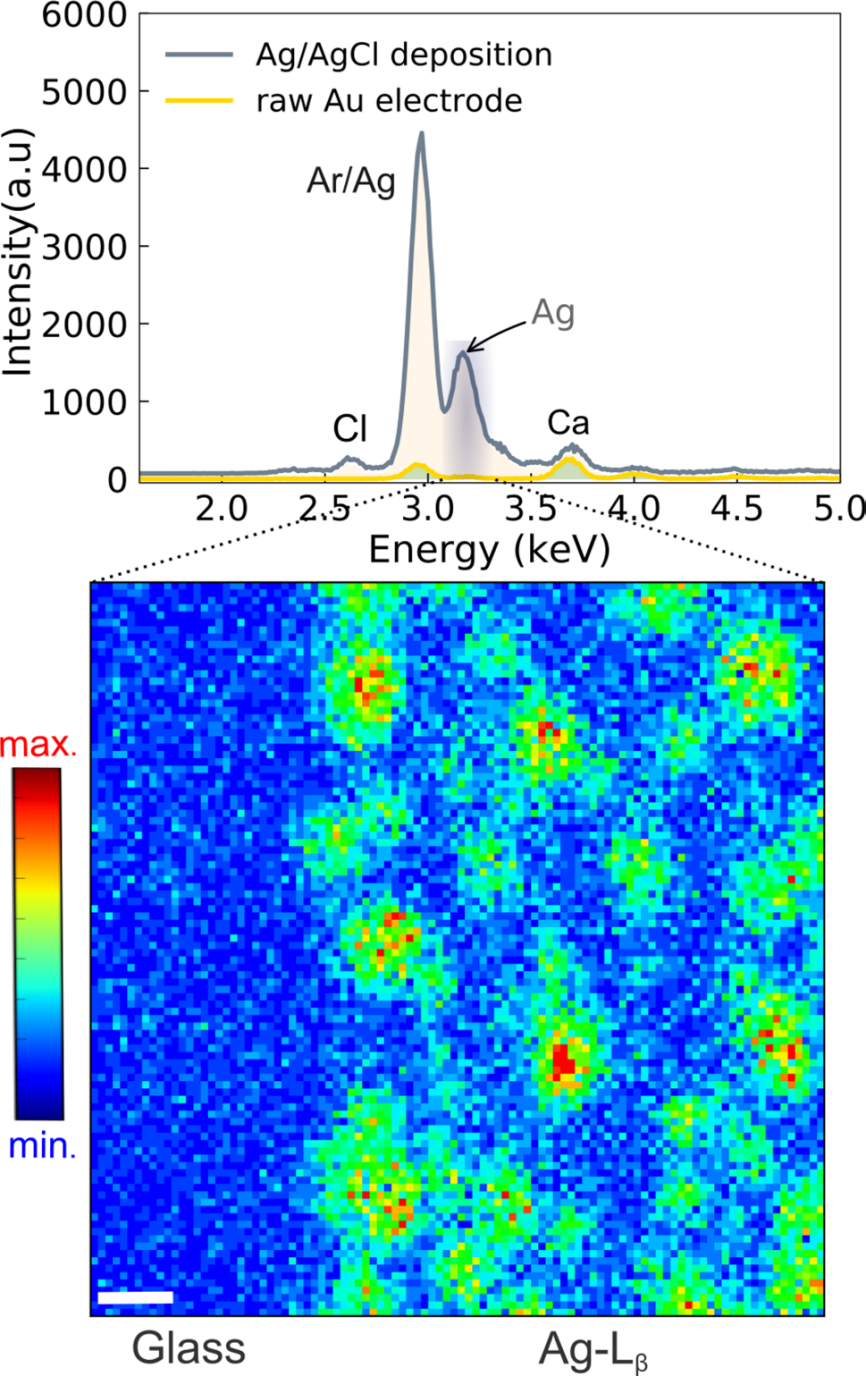
Preparation of Ag/AgCl film on a gold electrode: (top) Fluorescence spectra comparing a raw gold electrode with a modified one. It is perceptible the presence of the Ag and Cl after the electrodeposition as well as spurious lines (Ar and Ca); (Bottom) the 2D nanofluorescence map on the modified gold electrode revealing the sites where the Ag nucleated, forming islands-like morphology.The electrode was scanned with a nanobeam of 200 nm $\times$ 500 nm. The image has a pixel size of 500 nm and an acquisition time of 135 s per image (8 ms per pixel). The scale bar is 5 $\mu$m.

Besides the characterization of the device by observing the fluorescence of gold and chromium, nano-XRF was applied on an electrode covered with a silver/silver chloride (Ag/AgCl) film. The electrodeposited silver layer on the Au electrode was obtained by applying a negative potential in a solution containing silver ions at the desired Au electrode. Then, to form the AgCl, commercial sodium hypochlorite was pumped (50 $\mu {\hbox {L}^{\mathrm{-1}}}$) through the channel. Figure 6 shows the integrated spectrum (top) obtained over the area of 50 $\mu$m $\times$ 50 $\mu$m nm at steps of 500 nm (bottom panel). In this experiment, the electrode was positioned at the X-ray beam focus of about 200 $\times$ 500 ${{\hbox {nm}}^\mathrm{2}}$ (E=12.759 keV). In addition to Ag and Cl, minor peaks are seen associated with the glass substrate or contaminants. The 2D hyperspectral image (bottom) displays the Ag/AgCl film on the gold electrode obtained by selecting the energy corresponding to the Ag-$L_\beta$ (3.150 keV) as shown in the spectrum sum over the scanned area. The image reveals, with a submicrometer resolution, large islands of Ag on the electrode likely related to the mechanism of the nucleation process. The success in obtaining high fluorescence contrast, even at low energy (Ag-$L_\beta$), relies on the polyester film transparency, once at this energy, the attenuation is about 20 % (Supporting Information, Fig. S2). It is worth noting that this modification of the gold electrode provides an excellent internal reference of potential, being extremely important for future applications of the microfluidic cell with electrochemical reactions with precisely controlled applied potential, for example, *in situ * electrocatalysis experiments. This section’s results reinforce our device’s multifunctionality. It opens a broad path for further investigations using X-ray nanoprobe beamlines.

The device presented here was, at the first stage, validated using nano-XRF experiments. Nevertheless, it is applicable in different experiments involving X-rays, such as nanodifraction imaging, Bragg coherent diffractive imaging (BCDI), absorption, and X-ray excited optical luminescence. The reflection mode is an inherent drawback of the microfabrication geometry adopted. However, an advantage of our polyester-glass approach is the chemical resistance, which is high for a considerable range of aggressive organic solvents and acids and bases, where such stability is crucial for *in situ* experiments. Moreover, a large number of metals could be employed as electrodes. The experiments have demonstrated a satisfactory detection sensitivity at lower energies, including Cl at 2.6 keV in an air path of about 10 cm. Considering the measurements so far, we can improve the lower energy detection in further experiments by increasing the beam intensity and reducing air scattering. In addition, those mentioned techniques operate at relatively high angles. For example, for *in situ* diffraction experiments involving noble metals employed as catalysts (like Pt and Au), at beam energies from 7 to 10 keV, the q-vector range accessible covers the main reflections and operate at an incidence angle of about 20$^{\circ }$ or higher. Another reachable configuration is by rotating the device to access small angles, like GID (Grazing-incidence diffraction) and GISAXS (Grazing-incidence small-angle X-ray scattering experiments), where the incidence angle is above the glass total reflection. In such cases, taking into account the angle-dependent attenuation length (Supporting Information, Figs. S3, S4), fixes the incidence angle at a lower limit of 0.5-1$^{\circ }$ ( 9-12 keV), up to the limit established by the detection system, which is about 30$^{\circ }$ (or 2$\theta$=60$^{\circ }$) for the Tarumã/Carnaúba station. Still, it could be larger in other beamlines. Besides the parasitic scattering expected from the glass substrate at small angles, the sticker composed of NOA and polyester layer (Mylar) could contribute at significantly lower incidence angles (<0.5$^{\circ }$), nevertheless up to now, NOA has no data describing its scattering profile. As for comparison, an alternative design approach (^47,48^) employed a microfluidic cell for GISAXS experiments relying upon a cyclic olefin copolymer (COC). The cell has demonstrated good transparency to the X-rays, delivering a low background at angles close 0.5$^{\circ }$, but is not suitable to explore *in situ* electrochemistry experiments.

## Conclusions

In conclusion, we successfully developed a new glass-based microfluidic device sealing process using a thin polymer optimized for X-ray and/or optical measurements. The characterization reveals a versatile device exhibiting remarkable chemical resistance, which can operate as an electrochemical cell at room pressure and support high pressures when a volumetric flow ratio is needed. The device is very effective in reflection mode, showing high transparency in X-ray fluorescence experiments, proving to be suitable for *in situ * electrochemistry, and some envisaged *in vivo* experiments in liquids. It provides, in addition, the conditions for other X-ray techniques. Thus, it will be possible to exploit, besides *ex situ* experiments, advanced multi-techniques available at synchrotron beamlines.

## Materials and methods

The following chemical reactants were used during the different steps of the microfabrication process. A mix of sulfuric acid ${{\hbox {H}}_{\mathrm{2}}{{\hbox {SO}}_{\mathrm{4}}}}$ (Merck), water and hydrogen peroxide (H_2_O_2_/Merck) was the standard glass cleaning solution, acetone was used for the lift-toff of chromium and gold after sputtering deposition. Chromium mask etchant was purchased from TECHNIC. Photolithography process used AZ4620 photoresist and hexamethyldisilazane (HMDS) were purchased from Microchemicals GmbH (Ulm, Baden-Wurttemberg, Germany). A buffer solution of hydrofluoric acid (HF 49%, ${{\hbox {NH}}_{\mathrm{4}}F}$ 1.38 M and HCl 38%) and hydrofluoric acid (HF) 20% was purchased from Synth. Anhydrous potassium ferricyanide $({\hbox {K}_{3}[\hbox {Fe(CN)}_6]})$ and trihydrate potassium ferrocyanide $({\hbox {K}_{4}[\hbox {Fe(CN)}_6]})$ were purchased from J. T. Baker and Mallinckrodt, respectively. Potassium chloride (KCl) was purchased from Sigma-Aldrich. Hexane, n-propanol, BSA, KCl, and Bradford reagent were purchased from Sigma-Aldrich. The solutions were prepared in deionized water (purified in the Milli-Q system).

Commercial polyester polymer, called Mylar®, was used as sealing layer. Deposition of gold was performed by DC magnetron sputtering (AJA International inc.) and the chromium adhesion layer was prepared using an electron-beam deposition system (Angstrom engineering, evovac 046). The thickness of both metals, gold and chromium, was monitored using a quartz crystal microbalance. The size end depth of the channels was checked using a profilometer (Dektak 150) with a vertical resolution of 1 Å. The channels morphology and the quality of the electrodes were characterized using a stereoscopic microscope (Zeiss Discovery V12), and Agilent 34410A multimeter was used to measure the sheet resistance of the gold electrodes. A potentiostat/galvanostat (Autolab 302N) was used in the electrochemical tests. Spectrometer Thermo Scientific Multiskan for protein adsorption quantification. Microfluidic experiments have been done using a syringe pump (New Era Pump Systems, Inc., Farmingdale, NY, USA).

## Supplementary Information

Supplementary Information 1.

Supplementary Information 2.

Supplementary Information 3.
