# Supplementary material for: Development of a sticker sealed microfluidic device for in situ analytical measurements using synchrotron radiation
Source: Sci Rep. 2021 Dec 8;11:23671. doi: 10.1038/s41598-021-02928-2 (PMC8654830; doi:10.1038/s41598-021-02928-2)
Supplement: Supplementary file 2 — Supplementary Information 2. [file 41598_2021_2928_MOESM2_ESM.pdf]

Supporting Information for:

Development of a sticker sealed microfluidic device for in situ analytical measurements using synchrotron radiation

Itamar Neckel[1,], Lucas F. de Castro[2], Flavia Callefo[1], Verônica C. Teixeira[1], Angelo L. Gobbi[3], Maria H. Piazzetta[3], Ricardo A. G. de Oliveira[3], Renato S. Lima[3], Rafael A. Vicente[4], Douglas Galante[1], Helio C. N. Tolentino[1,*]*

[1]Brazilian Synchrotron Light Laboratory (LNLS), Brazilian Center for Research in Energy and Materials (CNPEM), Zip Code 13083-970, Campinas, São Paulo, Brazil.
[2]Instituto de Química, Universidade Federal de Goiás, Campus Samambaia, Goiânia, GO, 74690-900, Brazil
[3]Brazilian Nanotechnology National Laboratory (LNNano), Brazilian Center for Research in Energy and Materials (CNPEM), Zip Code 13083-970, Campinas, São Paulo, Brazil.
[4]Institute of Chemistry, University of Campinas, Campinas, São Paulo, Zip Code, 13083-970 Brazil

Corresponding authors: Itamar T. Neckel (itamar.neckel@lnls.br), Helio C. N. Tolentino (helio.tolentino@lnls.br)

A. Microfabrication method: channel preparation and electrode deposition

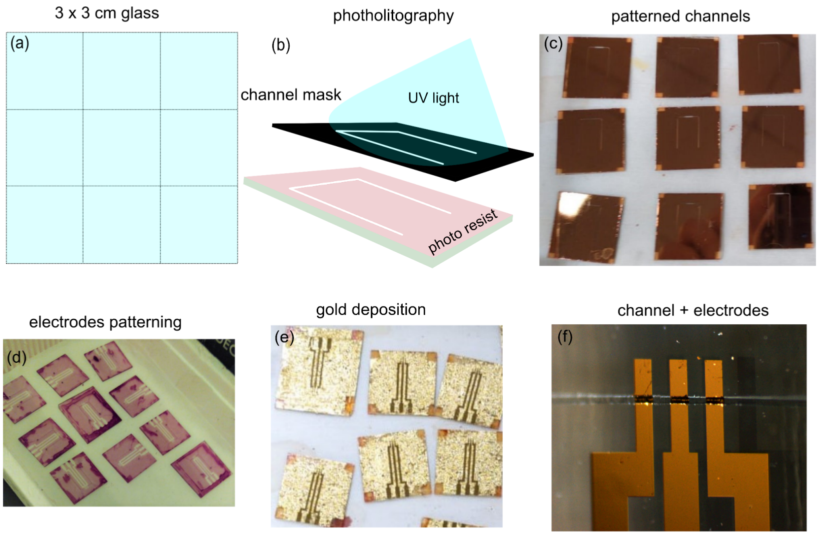

Figure S1: First steps of the microfabrication process. (a-c) The sequence of the channels preparation on glass and (d-e) Photolithography preparation of the electrodes and their deposition by magnetron sputtering on the channel. (f) Result for a non-sealed device (C1) with the three electrodes integrated on the channel.

B. X-ray transmission coefficient and attenuation length

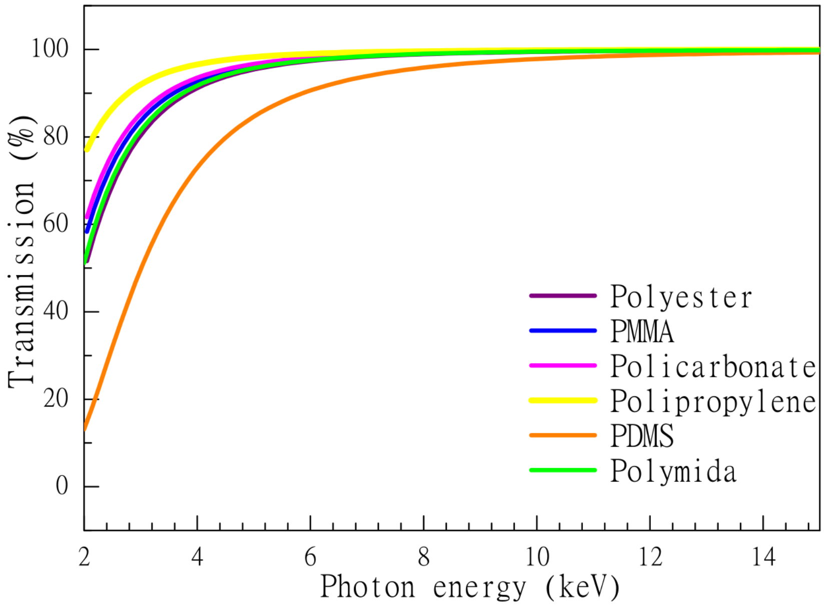

Figure S2: X-ray transmission of some polymers (12 μm thick) commonly applied in microfluidic devices. The photon energy range varies from 2 to 15 keV[1].

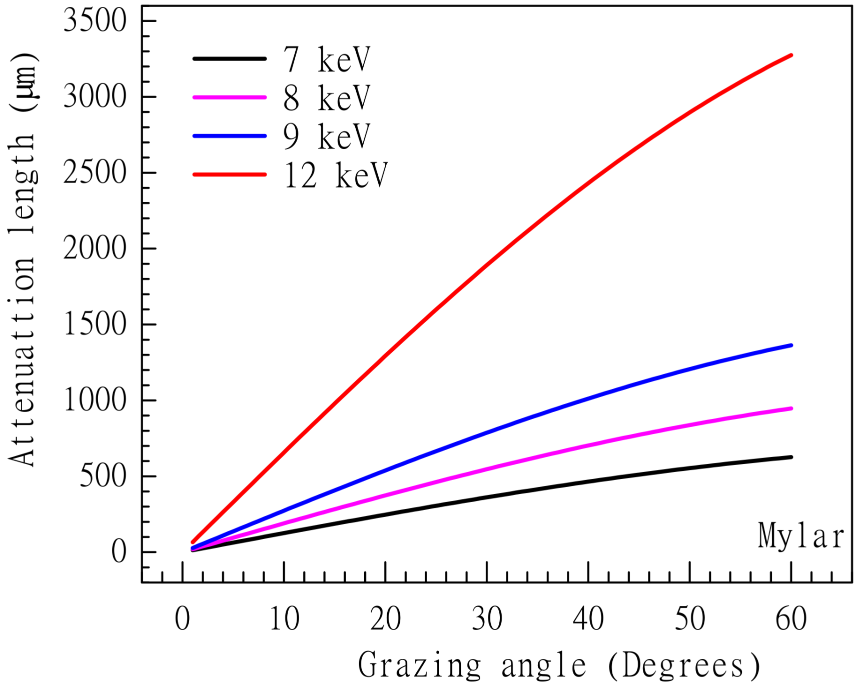

Figure S3: Angle-dependent attenuation length for the polyester film (Mylar). For lower energies (7-8 keV) the attenuation length at 1° is comparable to the polyester thickness (12 μm), meanwhile above 9 keV is twice or three times higher[1].

C. X-ray scattering path at grazing and high incidence angle

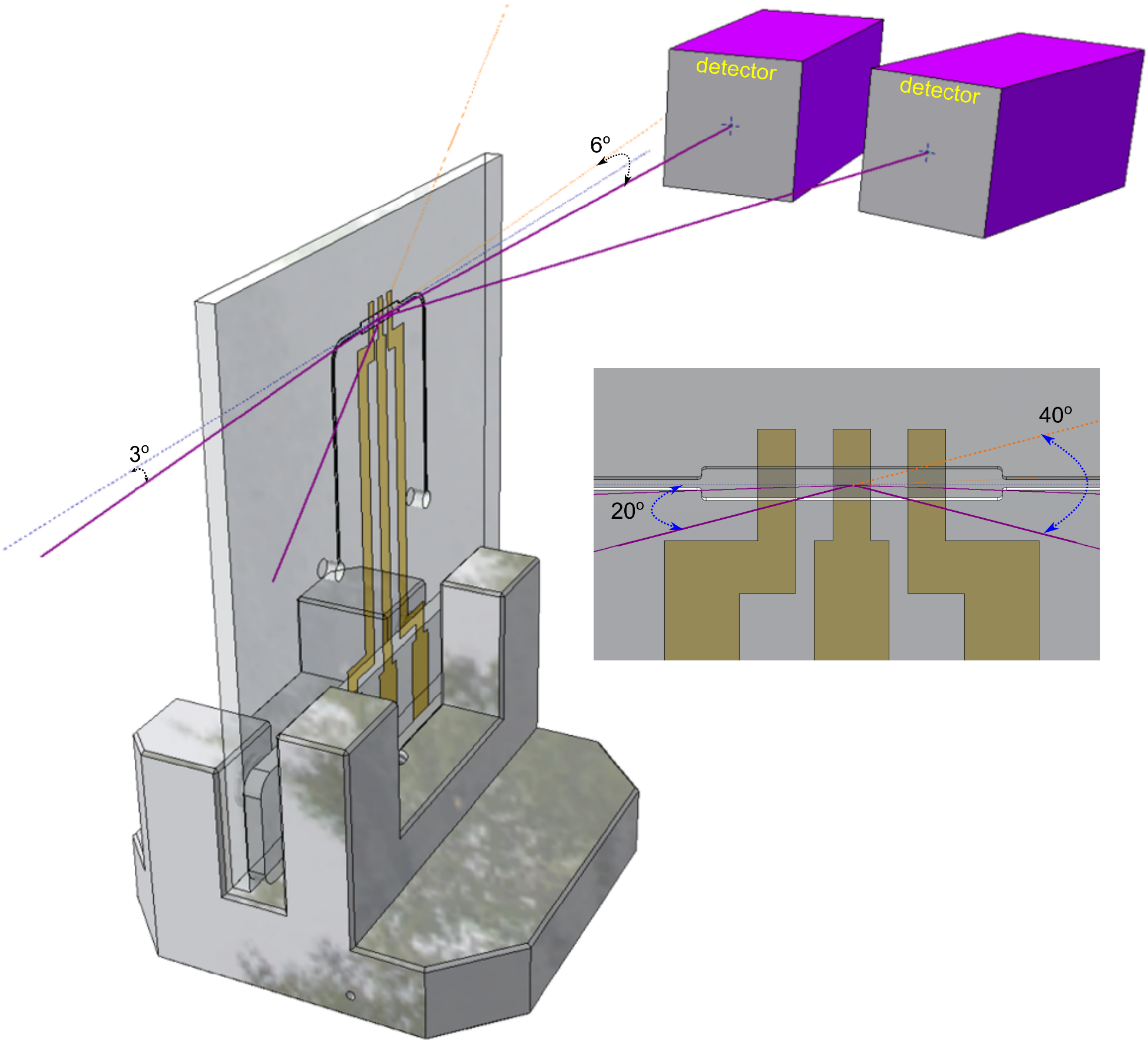

Figure S4: Schema of the scattering path in diffraction experiments at grazing and high angles. The beam enlarges following the channel direction.

References

[1] B.L. Henke, E.M. Gullikson, and J.C. Davis. *X-ray interactions: photoabsorption, scattering, transmission, and reflection at E=50-30000 eV, Z=1-92*, Atomic Data and Nuclear Data Tables Vol. 54 (no.2), 181-342 (July 1993)
