# Supplementary material for: Development of a sticker sealed microfluidic device for in situ analytical measurements using synchrotron radiation
Source: Sci Rep. 2021 Dec 8;11:23671. doi: 10.1038/s41598-021-02928-2 (PMC8654830; doi:10.1038/s41598-021-02928-2)
Supplement: Supplementary file 3 — Supplementary Information 3. [file 41598_2021_2928_MOESM3_ESM.pdf]

Video legend:

Video recorded after the complete microfabrication process. It was pumped water at 50 μl min^{-1} to validate the sealing process.
